# Correlation of Age, Gender, ABO Blood Group and Rh Factor in COVID-19 Infection: A Cross-Sectional Study in Udaipur, Rajasthan, India

**DOI:** 10.7759/cureus.57798

**Published:** 2024-04-08

**Authors:** Pramod B Jahagirdar, Kalpesh Vaishnav, Niharika A Sarathy, Harneet Singh, Gargi Nimbulkar, Karthikeyan Ramalingam

**Affiliations:** 1 Oral and Maxillofacial Pathology, Karnavati School of Research and Postdoctoral Studies, Gandhinagar, IND; 2 Oral and Maxillofacial Pathology, R. R. Dental College & Hospital, Udaipur, IND; 3 Prosthodontics, Crown and Bridge, Karnavati School of Dentistry, Gandhinagar, IND; 4 Oral Medicine and Radiology, Postgraduate Institute of Dental Sciences, Rohtak, IND; 5 Public Health Dentistry, R. R. Dental College & Hospital, Udaipur, IND; 6 Oral Pathology and Microbiology, Saveetha Dental College and Hospitals, Saveetha Institute of Medical and Technical Sciences, Saveetha University, Chennai, IND

**Keywords:** rhesus group, abo blood groups, abo blood-group system, blood testing, sars-cov-2, rh factor, pathogenicity, covid-19, coronavirus, abo

## Abstract

Background: The ABO blood group has long been recognized as a significant factor influencing susceptibility to infectious diseases. Numerous studies have explored the links between ABO blood types and both the likelihood of contracting COVID-19 and the severity of the infection, yielding conflicting results.

Aim: This study intends to determine the influence of age, gender, the ABO blood group, and Rh factor on the potential development of COVID-19 infection.

Methodology: A cross-sectional, observational study collected data including age, gender, the ABO blood group, and Rh factor from 80 healthcare professionals at R. R. Dental College and Hospital in Udaipur with a positive history of COVID-19 infection via Google Forms (Google LLC, Mountain View, California, United States). Chi-square statistics assessed the distribution of blood types and antibodies within the samples. Odds ratio (OR) assays were used to assess the probability of a certain blood type or Rh factor with version 21.0 of the IBM Statistical Package for Social Sciences (SPSS) for Windows (IBM Corp, Armonk, NY).

Results: In this study, the blood group type O was 45.2% (n = 33), type A was 21.9% (n = 16), type B was 24.7% (n = 18), and type AB was 8.2% (n = 6). Rh-positive samples were 87.7% (n = 64) and Rh-negative samples were 12.3% (n = 9). There was a statistically significant correlation between Type A (p = 0.001) and Type O (p = 0.049). Thirty-one participants (42.5%) were aged 20-30 years, 26 (35.6%) were aged 31-40 years, and 16 (21.9%) were aged 41-50 years. The statistical analysis revealed no statistically significant distinction among the age groups (p > 0.05).

Conclusion: The patients' gender, age, and concurrent disorders are crucial risk variables that determine the severity of severe acute respiratory syndrome-coronavirus 2 (SARS-CoV-2) infection. There is growing data indicating that the ABO blood group has a significant role in disease biology at physiological and biochemical levels. Hence, this study adds valuable information to strengthen and establish the potential role of factors, such as age and gender, in the possible pathogenicity of COVID-19 infection.

## Introduction

Severe acute respiratory syndrome-coronavirus 2 (SARS-CoV-2), the causative factor of COVID-19, emerged at the end of 2019 and spread rapidly across the globe [1]. A novel coronavirus, which shares structural similarities with the virus causing SARS, has been identified as the culprit. The medical, scientific, and public health communities have faced serious challenges as a result of the COVID-19 pandemic [2].

SARS-CoV-2 (a beta coronavirus) is classified under the subgenus *Sarbecovirus*. The fatality rates for severe pneumonia caused by SARS-CoV-2 and the Middle East severe respiratory syndrome caused by coronavirus (MERS-CoV) are 2.9%, 9.6%, and 36%, respectively [3]. There are four additional human coronaviruses, namely, the OC43, NL63, HKU1, and 229E, that commonly lead to a self-limiting illness characterized by mild symptoms. The examination and comparison of genomes reveal that SARS-CoV-2 possesses a strong preference for receptors that bind to angiotensin-converting enzyme 2 and a polybasic location for splitting at the S1/S2 spiked junction that influences infectivity [4]. SARS-CoV-2 infected individuals may display a range of symptoms, varying from minor to severe, while a considerable portion of the population remains silent. The symptoms most commonly reported are high temperature (83%), coughing (82%), and dyspnea (31%) [5].

The symptoms of the condition include a high body temperature, a dry cough, difficulty in breathing, tenderness in the muscles, tiredness, a sore throat, ageusia, and anosmia. Nevertheless, a significant number of affected individuals show no symptoms [6]. COVID-19 has a duration of incubation ranging from one to 14 days, with symptoms commonly appearing within three to seven days. There were numerous instances when the COVID-19 infection developed over 14 days to show any symptoms [7-11]. The International Society for the Study of Blood Transfusion (ISBT) has identified 34 distinct blood group types. ABO and Rh blood types are the most widely studied and implicated in treatment planning. The groups known as A, B, O, and AB types are ascertained based on the antigenic structure present on the surfaces of erythrocytes, whereas the Rh system is defined by the presence or lack of antigenic structure [8].

Various medical conditions, such as diabetes and hypertension, as well as sociodemographic characteristics, including sex, age, and race/ethnicity, have been confirmed as risk factors for catastrophic outcomes [10]. Emerging evidence suggests that the blood group known as ABO may influence the immunological foundation of SARS-CoV-2 infection. Group O provides immunity, while group A is linked to heightened vulnerability and severity of the illness [11]. The comprehension of the diverse groups of blood subtypes and their association with COVID-19 has the potential to contribute to the successful treatment and therapeutic approaches for the condition. Abuawwad et al. [9] observed that the subjects with the Rh+ type of blood had more likelihood of testing positive for SARS-CoV-2. Rh positivity and negativity play a distinct function in the development of COVID-19 infection.

Another issue investigated in COVID-19 is the chronological age of the sufferers. Elderly individuals and those with underlying health conditions are more susceptible to experiencing severe infection and have an increased likelihood of mortality due to the disease. According to a case series of 72,314 instances, the Chinese Centre for Prevention and Control of Diseases documented a fatal case rate of 8.0% among individuals over 70-79 years and 14.8% among individuals over 80 years [12]. Elderly individuals seem to be more vulnerable to the virus, with 75% of confirmed cases occurring in individuals aged 50 and above. While it is established that age significantly impacts the intensity of COVID-19 infection, the pathogenesis of this infection and its relation to the blood grouping system - ABO and Rh - remains unexplored.

Previous clinical research has demonstrated that females are less prone to viral infections and have lower cytokine production. Female patients exhibited increased macrophage and neutrophil activity, as well as enhanced antibody generation and response [13]. In-vivo studies showed that the angiotensin-converting enzyme 2 (ACE2) was more common in the kidneys of male patients compared to female patients. This could explain why COVID-19 is more likely to spread to males than to females [12].

Previous studies have examined the correlation between age, gender, and the ABO blood grouping system and COVID-19 in various countries, such as China, America, and Nigeria, by researchers like Zhao et al. [14], Szymanski et al. [15], and Kotila et al. [16]. Conflicting results exist regarding the infectivity and degree of COVID-19 infection in a certain blood group. In India, there is a lack of studies that confirm a connection between ABO blood groups and the COVID-19 infection. Increasing evidence indicates that the ABO blood type may play a role in disease biology at chemical and physiological levels. The study aims to investigate the potential impact of parameters, including years of age, gender, ABO blood grouping system, and Rh factor, on the development of COVID-19 infection in Rajasthan.

## Materials and methods

The Institutional Human Ethics Committee of R. R. Dental College and Hospital in Udaipur, Rajasthan, approved this study under reference number RR/IHEC/22. Data collection was conducted via a questionnaire, with no interventions involved. Initially, due to the paucity of data, a pilot study was conducted among 200 healthcare professionals to find out the prevalence of COVID-19 infection. It was self-reported COVID-19 infection status via Google Forms (Google LLC, Mountain View, California, United States). The results showed a self-reported prevalence of 4.5%. Based on this result, using a single proportion formula, the sample size was calculated to be 73 considering a 10% non-response rate. The data collected included age, gender, ABO blood group, and Rh Factor from healthcare professionals with a positive history of COVID-19 infection via Google Forms in Udaipur, Rajasthan.

The data were screened for the completeness of required data satisfied by 73 subjects and were included in the study (91.25% response rate among 80 recruited subjects). All the subjects with a positive COVID-19 RT-PCR report, ABO, and Rh blood group report were included in the study. Subjects in the age range between 20 years and 50 years were included. The questionnaires with incomplete information were excluded from the study.

Table 1 shows the questionnaire shared with the study subjects via Google Forms.

**Table 1:** Questions asked in the Google Forms questionnaire

S. No.	Parameters
1	Email address
2	Age
3	Gender
4	Past medical or systemic illness
5	Any ongoing medication for Systemic Illness (If yes, please specify)
6	ABO blood group
7	Rh factor
8	COVID report: a) RT-PCR, b) rapid antigen test
9	Severity: a) asymptomatic, b) mild, c) moderate, d) severe, e) critical
10	Admission to hospital
11	Recovery duration
12	Any laboratory parameter; C-reactive protein (CRP), IL-6, D-dimer, etc.
13	CT scan report

The collected data were entered into a Microsoft Excel worksheet (Microsoft Corporation, USA). The data analysis was performed utilizing the IBM Statistical Program for the Social Sciences (statistical analysis for Microsoft Windows, version 21.0, IBM Corporation, Armonk, NY, USA). The analysis of categorical data involved the utilization of frequencies and percentages. The distributions of blood groups and autoantibodies among the samples were assessed using a Chi-squared test with a significant p-value of less than 0.05. The study utilized odds ratio (OR) testing to assess the probability of a certain blood type or Rh factor.

## Results

According to the responses obtained via Google Forms, the mean age of the subjects who answered the questionnaire was 33 years; 61 subjects (83.6%) were females and 48 subjects (66.1%) were not associated with systemic illness. Thirty-three subjects had hypertension (4.8%) as the most prevalent systemic ailment, followed by two subjects with asthma (1.6%). Seventy subjects (95.9%) had a positive RT-PCR report for COVID-19, 32 subjects (43.8%) stated that they were asymptomatic, and 31 subjects (42.5%) gave a history of mild symptoms. Nine subjects (12.3%) stated that the duration of their illness lasted for five days. Forty-seven subjects (65%) did not get any further laboratory investigations, and 69 subjects (95%) did not get a computed tomography (CT) examination. Thirty-one individuals (42.5%) were aged 20-30 years, 26 participants (35.6 percent) were of the age range 31-40 years, and 16 participants (21.9%) were in the age range of 41-50 years. The difference in age distribution did not appear to be significantly different (χ2 = 4.795, p = 0.091 NS) (Table 2).

**Table 2:** Age distribution of the study samples NS - Not significant

Age group	Number	Percentage	P value
20-30 years	31	42.5	χ2=4.795 p=0.091 NS
31-40 years	26	35.6	
41-50 years	16	21.9	
Total	73	100	

There were six (17.6%), 11 (32.4%), 14 (41.2%), and three (8.8%) males with A, B, O, and AB blood groups correspondingly. For females, there were eight (20.5%), ninw (23.1%), 19 (48.7%), and three (7.7%) with the same blood groups. No statistically significant difference was observed in the gender distribution (p > 0.05) (Table 3).

**Table 3:** Gender and ABO group distribution in the study samples NS - Not significant

Blood group	Males		Females		p-value
	Number	Percentage	Number	Percentage	
A	6	17.6	8	20.5	0.755 NS
B	11	32.4	9	23.1	0.377 NS
O	14	41.2	19	48.7	0.523 NS
AB	3	8.8	3	7.7	0.865 NS
Total	34	100	39	100	-

Group O exhibited the highest frequency among the COVID-19-infected samples followed by groups B, group A, and group AB. In the COVID-19-infected samples, nine persons (12.3%) were Rh-negative, and 64 participants (87.7%) were Rh-positive (Table 4).

**Table 4:** Distribution of ABO blood groups and COVID-19 infection

Blood group	COVID-19 infection observed in our study	
	Number	Percentage
A	16	21.9
B	18	24.7
O	33	45.2
AB	6	8.2
Total	73	100
Rh-negative	9	12.3
Rh positive	64	87.7
Total	73	100

If an individual had blood group A, their likelihood of contracting COVID-19 was 1.948 times higher compared to the overall distribution of blood types in the population. The observed disparity exhibited statistical significance, as shown by Pearson's p-value of 0.001 or lower. The probability of an individual with blood type B developing COVID-19 was 0.726 (CI = 0.485-1.806), but this finding did not reach statistical significance (p = 0.068). The COVID-19 infection chances ratio for individuals with blood group O was 1.355 (CI = 0.985-1.866), indicating a statistically significant distinction (p = 0.049). The OR for COVID-19 infection in individuals with the AB blood group was 0.857 (95% CI: 0.457-1.606), but it did not reach statistical significance (p = 0.411). Subjects who were RH-positive had an infection odds of 0.534 (CI = 0.201-11.421), whereas those who were Rh-negative had odds of 0.645 (0.358-1.761). According to the statistical analysis, the observed disparity in infection probabilities among both groups did not reach statistical significance, as evidenced by Pearson's values of 0.113 and 0.097, respectively (Table 5).

**Table 5:** Comparison of ABO blood groups and COVID-19 infection *Statistically significant with p-value < 0.05. NS- not significant, OR - odds ratio, CI - confidence interval

Blood type	χ2	OR	95% CI	P-value
A	16.686	1.948	1.417-2.678	>0.001*
B	2.775	0.726	0.485-1.806	0.068 NS
O	3.325	1.355	0.985-1.866	0.049*
AB	0.259	0.857	0.457-1.606	0.411 NS
Rh-positive	2.312	0.534	0.201-1.421	0.113 NS
Rh-negative	2.532	0.645	0.358-1.761	0.097 NS

## Discussion

All potential mechanisms linking the ABO type of blood classes and COVID-19 are summarized in Figure 2.

**Figure 1:**
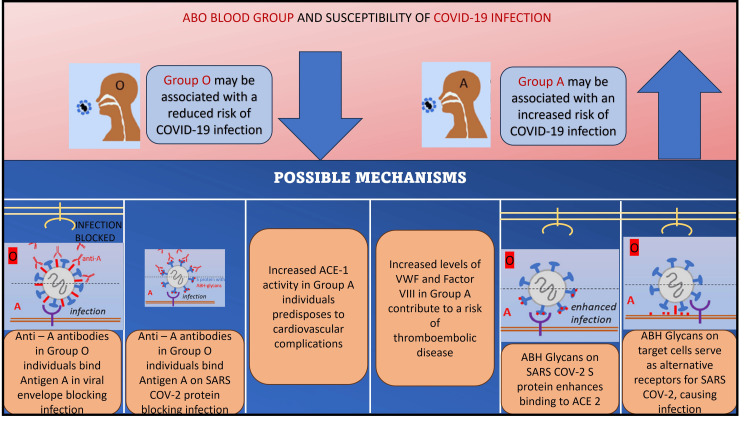
Schematic representation of possible mechanisms in the pathogenesis of COVID-19 in different ABO blood groups. ACE - angiotensin-converting enzyme, VWF - Von Willebrand factor, ABH - blood group antigens, COVID - coronavirus disease, SARS - severe acute respiratory syndrome Image credit: Pramod J and Niharika S

The correlation between COVID-19 and the blood types ABO and Rh has been reported in scientific literature [17,18]. The ABO blood group system has two antigens, namely, A and B [18]. The genetic sequence responsible for encoding the antigenic protein is located on chromosomes 9q34.1 and 9q34.2. The genetic composition of the system consists of three distinct alleles (A, B, and O) and four different traits (A, B, O, and AB) [19].

**Figure 2:**
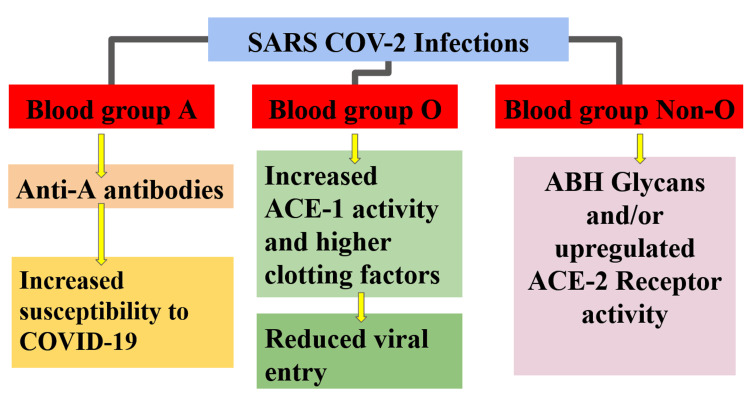
Proposed mechanisms for ABO and COVID-19 infection ACE - angiotensin-converting enzyme, ACE2R - angiotensin-converting enzyme-2 receptor, ABH - blood group antigens, COVID - coronavirus disease, SARS - severe acute respiratory syndrome Image credit: Karthikeyan Ramalingam, Pramod J, and Niharika S

Batool et al., Storry et al., and Cheng et al. examined the correlation between ABO blood classes and various viral infections [20-22]. The blood group known as ABO has been linked to viral illnesses. The findings of a study suggest that individuals with the O blood group have reduced vulnerability to hepatitis B, C, HIV, syphilis, and malaria [20]. The proposed mechanisms for ABO and COVID-19 infection are shown in Figure 2.

The application of hemagglutination tests is employed for the identification of glycosylated hemoglobin (HBGAs) on the external surface of erythrocyte membranes. Complex chemicals influence disease progression through the activity of natural antibodies [21]. Research has extensively investigated the connection between blood group ABO and other diseases, including viral illnesses. Cheng et al. forecasted the vulnerability to infection with the SARS-CoV in Hong Kong based on the ABO blood group systems. The findings of the study indicated that hospital personnel who possess the O blood type have a decreased susceptibility to infections in comparison to persons with non-O blood groups [22]. This study showed that blood types A (45.2%) and B (24.7%) are the most common blood types among COVID-19 patients. There were 64 patients with Rh-positive blood type (87.7%) and nine patients with Rh-negative blood type (12.3%). Our findings aligned with Zhao et al., indicating that those with blood type A had more likelihood of contracting COVID-19 [14]. Muniz-Diaz et al.'s [23] investigation confirmed a link between the blood group known as ABO and the likelihood of contracting COVID-19. Individuals belonging to group A had a higher susceptibility to this viral infection, whereas individuals in group O displayed a lower susceptibility [24].

Rana et al. conducted a study on the Indian population, which revealed that individuals having blood types A, B, and Rh+ had a greater vulnerability to COVID-19 infection. Conversely, individuals with blood categories AB, O, and Rh- demonstrated a significantly reduced chance of catching COVID-19 [8]. We observed a statistically significant correlation only between blood types A and O with COVID-19 infections. Comparisons with other blood groups were not statistically significant. This discrepancy in the outcome may be due to variances in the prevalence of different groupings of blood types in the study group and the sample size.

Antigens belonging to the ABO blood group are found on red blood cells, epithelial cells in the digestive and respiratory tracts, and endothelial cells beneath blood arteries, which are capable of producing ABH carbohydrate structures [25]. ABO blood grouping studies have been identified in the dental pulp tissue and secretor status was also noted in salivary samples [26,27]. The S protein of virions produced by individuals of type A may be adorned with A carbohydrate epitopes, while those produced by type B individuals could display B carbohydrate epitopes. Antibodies from blood groups O, B, and A persons can attach to specific sites on the S protein, which is a component of viral particles, inhibiting its binding to ACE2 protein receptors on host cell membranes and thereby blocking infection. It is theorized that anti-A antibodies and/or anti-B antibodies could attach to A and/or B antigens on the viral envelope, potentially blocking the infection of target cells. In other words, these antibodies may act as viral-neutralizing antibodies [28].

Gerard et al. reported that the presence of IgG anti-A antibodies in the serum has more significance on the correlation of COVID-19 with ABO blood groups [28]. Zaidi et al. [29] reported that blood group A had a higher risk and blood type O had a lower risk for COVID-19. Rana et al. [8] reported that compared to female individuals with the same blood type, male patients with blood group B had a higher risk of having COVID-19 infection. Muniz-Diaz et al. reported that individuals over the age of 60 were highly susceptible to COVID-19, with more than 90% of fatalities occurring in this age group [24]. We did not find any gender variations among our study samples.

The literature suggests that the ABO group could play a role in the prognostic value of COVID-19. The use of convalescent plasma for COVID-19 treatment has been advocated, and ABO grouping will play a significant role in such situations [29]. Using data from NYP/CUIMC, Zietz et al. found moderately increased infection prevalence among non-O blood types and Rh-positive individuals. Intubation risk was increased among AB and B types and decreased among A and Rh-negative types. The risk of death was slightly increased among type AB individuals and was decreased among type A, B, and Rh-negative individuals. All estimates were adjusted for patient ancestry using self-reported race and ethnicity [30]. Pereira et al. reported that three major hypotheses emerged. SARS-CoV-2 could carry ABO(H)-like structures in its envelope glycoproteins and would be asymmetrically transmitted due to a protective effect of the ABO antibodies, ABH antigens could facilitate SARS-CoV-2 interaction with the host cells, and the association of non-O blood types with higher risks of thromboembolic events could confer COVID-19 patients with blood type O a lower risk of severe outcomes [31].

Limited information is available about ABO groups and COVID-19 among the Indian population. Our investigation did not have statistically significant results. Additional research with an increased sample size across several centers is required to validate our findings. A limitation is that this study was performed in a single city within Rajasthan. Further multi-centric studies with regional and ethnic variations are the key to further exploration of this association. The degree of severity of COVID-19 infection may be linked to ABO blood groups and might be verified by biomarkers, such as CRP, IL-6, and D-dimers, in the future.

## Conclusions

Patients' age, gender, and comorbidities are crucial risk variables that can predict the severity of SARS-CoV-2 infection. There are growing data indicating that the ABO blood group has a clear impact on disease biology at physiological and biochemical levels. The study evaluated the potential influence of age and sex on COVID-19 infection. We have observed higher infection among group O and group A individuals but other groups did not attain statistical significance. We did not observe any gender variations in our study. It has to be validated with multi-centric studies.

## References

[1] Yang W, Sirajuddin A, Zhang X, Liu G, Teng Z, Zhao S, Lu M (2020). The role of imaging in 2019 novel coronavirus pneumonia (COVID-19). Eur Radiol.

[2] de Wit E, van Doremalen N, Falzarano D, Munster VJ (2016). SARS and MERS: recent insights into emerging coronaviruses. Nat Rev Microbiol.

[3] Wang C, Horby PW, Hayden FG, Gao GF (2020). A novel coronavirus outbreak of global health concern. Lancet.

[4] Andersen KG, Rambaut A, Lipkin WI, Holmes EC, Garry RF (2020). The proximal origin of SARS-CoV-2. Nat Med.

[5] Ciotti M, Ciccozzi M, Terrinoni A, Jiang WC, Wang CB, Bernardini S (2020). The COVID-19 pandemic. Crit Rev Clin Lab Sci.

[6] Rodriguez-Morales AJ, Cardona-Ospina JA, Gutiérrez-Ocampo E (2020). Clinical, laboratory and imaging features of COVID-19: a systematic review and meta-analysis. Travel Med Infect Dis.

[7] Huang C, Wang Y, Li X (2020). Clinical features of patients infected with 2019 novel coronavirus in Wuhan, China. Lancet.

[8] Rana R, Ranjan V, Kumar N (2021). Association of ABO and Rh blood group in susceptibility, severity, and mortality of coronavirus disease 2019: a hospital-based study from Delhi, India. Front Cell Infect Microbiol.

[9] Abuawwad MT, Taha MJ, Abu-Ismail L (2022). Effects of ABO blood groups and RH-factor on COVID-19 transmission, course and outcome: a review. Front Med (Lausanne).

[10] Wu Z, McGoogan JM (2020). Characteristics of and important lessons from the coronavirus disease 2019 (COVID-19) outbreak in China: summary of a report of 72 314 cases from the Chinese Center for Disease Control and Prevention. JAMA.

[11] Lithander FE, Neumann S, Tenison E (2020). COVID-19 in older people: a rapid clinical review. Age Ageing.

[12] Kopel J, Perisetti A, Roghani A, Aziz M, Gajendran M, Goyal H (2020). Racial and gender-based differences in COVID-19. Front Public Health.

[13] Wiersinga WJ, Rhodes A, Cheng AC, Peacock SJ, Prescott HC (2020). Pathophysiology, transmission, diagnosis, and treatment of coronavirus disease 2019 (COVID- 19): a review. JAMA.

[14] Zhao J, Yang Y, Huang H (2021). Relationship between the ABO blood group and the coronavirus disease 2019 (COVID-19) susceptibility. Clin Infect Dis.

[15] Szymanski J, Mohrmann L, Carter J (2021). ABO blood type association with SARS-CoV-2 infection mortality: a single-center population in New York City. Transfusion.

[16] Kotila TR, Alonge TO, Fowotade A, Famuyiwa OI, Akinbile AS (2021). Association of the ABO blood group with SARS-CoV-2 infection in a community with low infection rate. Vox Sang.

[17] Aljanobi GA, Alhajjaj AH, Alkhabbaz FL, Al-Jishi JM (2020). The relationship between ABO blood group type and the COVID-19 susceptibility in Qatif Central Hospital, Eastern Province, Saudi Arabia: a retrospective cohort study. Open J Intern Med.

[18] Amundadottir L, Kraft P, Stolzenberg-Solomon RZ (2009). Genome-wide association study identifies variants in the ABO locus associated with susceptibility to pancreatic cancer. Nat Genet.

[19] Vasan SK, Rostgaard K, Majeed A (2016). ABO blood group and risk of thromboembolic and arterial disease: a study of 1.5 million blood donors. Circulation.

[20] Batool Z, Durrani SH, Tariq S (2017). Association of ABO and Rh blood group types to hepatitis B, hepatitis C, HIV and syphilis infection, a five year ’experience in healthy blood donors in a tertiary care hospital. J Ayub Med Coll Abbottabad.

[21] Storry JR, Olsson ML (2009). The ABO blood group system revisited: a review and update. Immunohematology.

[22] Cheng Y, Cheng G, Chui CH (2005). ABO blood group and susceptibility to severe acute respiratory syndrome. JAMA.

[23] Muñiz-Diaz E, Llopis J, Parra R (2021). Relationship between the ABO blood group and COVID-19 susceptibility, severity and mortality in two cohorts of patients. Blood Transfus.

[24] Guillon P, Clément M, Sébille V, Rivain JG, Chou CF, Ruvoën-Clouet N, Le Pendu J (2008). Inhibition of the interaction between the SARS-CoV spike protein and its cellular receptor by anti-histo-blood group antibodies. Glycobiology.

[25] Goel R, Bloch EM, Pirenne F (2021). ABO blood group and COVID-19: a review on behalf of the ISBT COVID-19 Working Group. Vox Sang.

[26] Das M, Banerjee A, Samanta J, Bhunia BB, Mozumder S, Ramalingam K (2023). ABO blood grouping and rhesus factor determination from dental pulp tissue: a forensic research. Cureus.

[27] Rajawat G, Ramalingam K, Pareek R, Singh G, Narula H, Aggarwal A (2023). Assessment of salivary ABO blood group antigens and secretor status in Sriganganagar, Rajasthan: a correlational analysis of 300 samples. Cureus.

[28] Gérard C, Maggipinto G, Minon JM (2020). COVID-19 and ABO blood group: another viewpoint. Br J Haematol.

[29] Zaidi FZ, Zaidi AR, Abdullah SM, Zaidi SZ (2020). COVID-19 and the ABO blood group connection. Transfus Apher Sci.

[30] Zietz M, Zucker J, Tatonetti NP (2020). Testing the association between blood type and COVID-19 infection, intubation, and death. medRxiv.

[31] Pereira E, Felipe S, de Freitas R (2022). ABO blood group and link to COVID-19: a comprehensive review of the reported associations and their possible underlying mechanisms. Microb Pathog.

